# A clinically applicable molecular classification of oncocytic cell thyroid nodules

**DOI:** 10.1530/ERC-23-0047

**Published:** 2023-08-03

**Authors:** Elizabeth J de Koster, Willem E Corver, Lioe-Fee de Geus-Oei, Wim J G Oyen, Dina Ruano, Abbey Schepers, Marieke Snel, Tom van Wezel, Dennis Vriens, Hans Morreau

**Affiliations:** 1 Department of Medical Imaging, Nuclear Medicine, Radboud University Medical Centre, Nijmegen, the Netherlands; 2 Department of Radiology, Section of Nuclear Medicine, Leiden University Medical Center, Leiden, the Netherlands; 3 Department of Pathology, Leiden University Medical Center, Leiden, the Netherlands; 4 Biomedical Photonic Imaging Group, University of Twente, Enschede, the Netherlands; 5 Department of Radiology and Nuclear Medicine, Rijnstate Hospital, Arnhem, the Netherlands; 6 Department of Biomedical Sciences and Humanitas Clinical and Research Centre, Department of Nuclear Medicine, Humanitas University, Milan, Italy; 7 Department of Surgery, Leiden University Medical Center, Leiden, the Netherlands; 8 Department of Medicine, Division of Endocrinology, Leiden University Medical Center, Leiden, the Netherlands

**Keywords:** oncocytic cell, thyroid, genome haploidization, copy number alterations, molecular diagnostics

## Abstract

Whole chromosome instability with near-whole genome haploidization (GH) and subsequent endoreduplication is considered a main genomic driver in the tumorigenesis of oncocytic cell thyroid neoplasms (OCN). These copy number alterations (CNA) occur less frequently in oncocytic thyroid adenoma (OA) than in oncocytic carcinoma (OCA), suggesting a continuous process. The current study described the CNA patterns in a cohort of 30 benign and malignant OCN, observed using a next-generation sequencing (NGS) panel that assesses genome-wide loss of heterozygosity (LOH) and chromosomal imbalances using 1500 single-nucleotide polymorphisms (SNPs) across all autosomes and the X chromosome in DNA derived from cytological and histological samples. Observed CNA patterns were verified using multiparameter DNA flow cytometry with or without whole-genome SNP array analysis and lesser-allele intensity-ratio (LAIR) analysis. On CNA–LOH analysis using the NGS panel, GH-type CNA were observed in 4 of 11 (36%) OA and in 14 of 16 OCA (88%). Endoreduplication was suspected in 8 of 16 (50%) OCA, all with more extensive GH-type CNA (*P <* 0.001). Reciprocal chromosomal imbalance type CNA, characterized by (imbalanced) chromosomal copy number gains and associated with benign disease, were observed in 6 of 11 (55%) OA and one equivocal case of OCA. CNA patterns were different between the histopathological subgroups (*P <* 0.001). By applying the structured interpretation and considerations provided by the current study, CNA–LOH analysis using an NGS panel that is feasible for daily practice may be of great added value to the widespread application of molecular diagnostics in the diagnosis and risk stratification of OCN.

## Introduction

Thyroid oncocytic cells are follicular-derived oncocytic cells that are characterized by granular, eosinophilic cytoplasm due to an abundance of mitochondria. Their nuclei are enlarged and rounded, with prominent nucleoli (Wong *et al.* 2021). Before the introduction of the 2023 WHO classification referred to as Hürthle cell neoplasms, oncocytic cell neoplasms (OCN) are defined as thyroid neoplasms composed of >75% oncocytic cells and include oncocytic thyroid adenoma (OA) and carcinoma (OCA) (Lloyd *et al.* 2017, Baloch *et al.* 2022, WHO Classification of Tumours Editorial Board 2022). OCN are distinct from other types of thyroid nodules or differentiated thyroid carcinoma in their biological and clinical behavior. OCA, in particular widely invasive OCA, typically show more aggressive behavior and less favorable prognosis than their non-oncocytic cell follicular counterparts, including higher rates of extra-thyroidal extension, radioiodine-refractory disease, distant metastases, and mortality (Goffredo *et al.* 2013, Zhou *et al.* 2020). Historically defined as a subtype of follicular thyroid carcinoma, the 2017 WHO classification has recognized OCA as a completely separate entity in follicular neoplasia (Lloyd *et al.* 2017). Distinguishing benign from malignant OCN may be complex: cytological differentiation is not possible as (histopathological) assessment of capsular and vascular invasion is required, interobserver variability is observed, and metastases of OCN with an initial morphological diagnosis of OA have been described (Grant *et al.* 1988, Boronat *et al.* 2013, Cibas & Ali 2017, Thodou *et al.* 2021). In addition, oncocytic cell metaplasia/hyperplasia in the thyroid should be clearly distinguished from true OCN, as it is different in both origin and genetic alterations. It may occur in lymphocytic thyroiditis, oncocytic variant papillary thyroid carcinoma, medullary thyroid carcinoma, parathyroid lesions, and non-thyroid metastasis (Asa & Mete 2021, Thodou *et al.* 2021).

Whole-chromosome instability is a main characteristic of tumorigenesis in OCN. The process might be driven by a continuous redox imbalance due to the accumulation of malfunctioning mitochondria causing mitotic errors, leading to near-whole genome haploidization (GH, i.e., consistent with an A0 genotype) with subsequent endoreduplication (i.e., genome doubling, an AA genotype, or multiple thereof). These copy number alterations (CNA) seem to occur stepwise and in patterns in the progression from OA to OCA (Corver *et al.* 2018). More extensive loss of heterozygosity (LOH) and endoreduplication are associated with the progression of the disease and worse prognosis (Wada *et al.* 2002, Corver *et al.* 2012, 2014, Ganly *et al.* 2018, Gopal *et al.* 2018, Jalaly & Baloch 2020, Doerfler *et al.* 2021). Corver *et al.* previously described the LOH of chromosomes 1, 2, 8, 9, 18, and 22 as early events and the additional loss of chromosomes 2, 3, 6, 11, 14–16, and 21 as later events indicating progression of the disease, with the loss of chromosomes 1–4, 6, and 11 as the minimum signature of OCA (Corver *et al.* 2018). Similar patterns were observed by Ganly *et al.*, who described the LOH of chromosomes 2, 9, 11, and 18 as early events (Ganly *et al.* 2018). The LOH of chromosome 7 is never observed, likely due to maternal- and paternal-imprinted genes important for survival (Boot *et al.* 2016). Chromosomes 5, 12, and 20 are also frequently spared (Corver *et al.* 2018, Ganly *et al.* 2018). To a lesser extent, these characteristic CNA patterns are also described in part of the OA (Tallini *et al.* 1999, Wada *et al.* 2002, Stankov *et al.* 2004, Corver *et al.* 2018, Doerfler *et al.* 2021). Other, reciprocal patterns of imbalanced chromosomal gains, consistent with genotype AAB, have also been observed in OA (Wada *et al.* 2002, Corver *et al.* 2018). In addition, driver point mutations (e.g., TERT promoter or TP53) have also been described in OCA (Ganly *et al.* 2013, Corver *et al.* 2018, Ganly *et al.* 2018, Jalaly & Baloch 2020, Kumari *et al.* 2020, Santana *et al.* 2020, Doerfler *et al.* 2021).

To advance the diagnostic workup and risk stratification of OCN, the development of a molecular test that is both accurate and feasible in everyday clinical practice is crucial. The methods previously used for CNA and LOH (CNA–LOH) analysis by Corver *et al.* and Ganly *et al.* are highly accurate and informative but time-consuming, costly, and thus not fit for everyday application (Corver *et al.* 2008, 2012, 2018, Ganly *et al.* 2018). Corver *et al.* combined multiparameter DNA flow cytometry with whole-genome single-nucleotide polymorphism (SNP) array analysis (using 6000 to >200,000 SNPs) (Corver *et al.* 2008, 2018). The algorithm used is referred to as lesser-allele intensity-ratio (LAIR) analysis, which incorporates the DNA index of the G_0_G_1_ tumor cell fraction into the CNA–LOH analysis (Corver *et al.* 2008), validated by fluorescence in-situ hybridization (FISH) (Corver *et al.* 2012). Ganly *et al.* combined computational fraction and allele-specific copy-number estimates from tumor sequencing and FISH (Kushchayeva *et al.* 2008, Ganly *et al.* 2018). Doerfler *et al.* used the commercial ThyroSeq® v3, which is applied on a large scale in clinical practice in the USA (Doerfler *et al.* 2021, Hu *et al.* 2022). They distinguished two main types of CNA: a GH-type characterized by widespread chromosomal losses and a focal chromosomal type characterized by random losses and gains of chromosomal regions. Unfortunately, full information regarding their interpretation of the CNA analysis, distinguished chromosomal patterns, and details on the underlying chromosomal alterations were not presented (Nikiforova *et al.* 2018, Doerfler *et al.* 2021).

The current study aimed to implement a clinically applicable test using a limited 1500 SNP next-generation sequencing (NGS) panel. We described the CNA patterns that we observed in benign and malignant OCN and assessed them in a clinical cohort of OCN, with certain focus on the methodological and bioinformatical aspects. We highlighted two cases to illustrate the added value of CNA–LOH analysis in clinical practice. Finally, we provided considerations for the structured interpretation of the CNA–LOH analysis results using this NGS panel.

## Materials and methods

### Study design and case selection

For the current retrospective study, pseudonymized pathology records were reviewed to identify cases with an OCN of the thyroid in which molecular analysis including CNA–LOH analysis was performed on the primary tumor during clinical practice at our tertiary care center between May 1, 2020, and December 31, 2021. Patients were only eligible for inclusion if the diagnosis of an oncocytic cell lesion was confirmed by histopathology, including nodular hyperplasia with oncocytic cell metaplasia (NH-H), OA, and OCA. Prior to inclusion in the current study, all histopathological diagnoses were made or reviewed by an experienced thyroid pathologist (HM) in accordance with the WHO classification (5th edition), including a morphological assessment and immunohistochemistry of H&E sections (WHO Classification of Tumours Editorial Board 2022). Ethical study review was waived by the medical ethical review committee Leiden the Hague Delft (no. G21.167). No informed consent was required.

### Data collection

From the pathology records, we recorded cytopathological and histopathological characteristics. Patient demographics and clinical, radiological, and surgical characteristics were collected from the patient medical records. Thyroid cytopathology was described using the Bethesda classification (Cibas & Ali 2017). The Thyroid Imaging Reporting and Data System (TIRADS) classification was infrequently reported, and it was considered inappropriate to retrospectively reassess stored ultrasound captures, as ultrasound is a dynamic technique (Tessler *et al.* 2017). Follow-up data were updated until October 1, 2022.

### Molecular analysis

**Figure 1:**
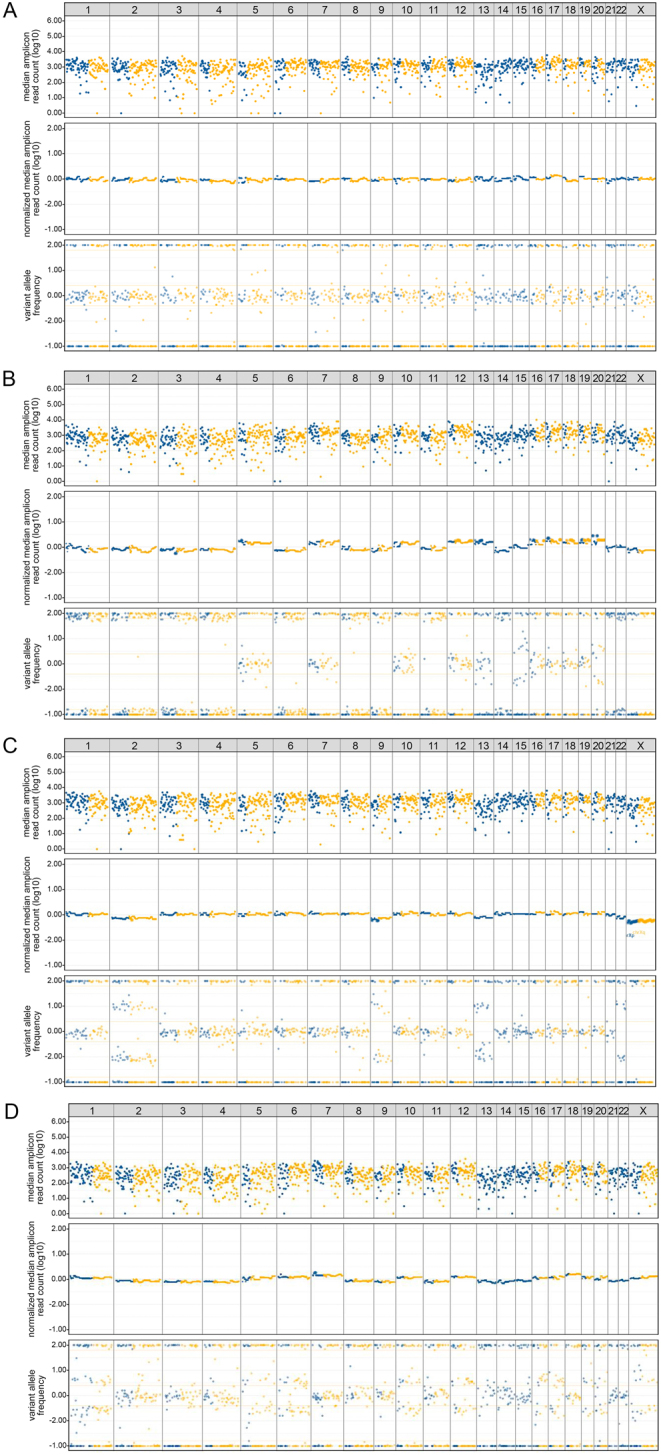
The main CNA patterns, observed by CNA–LOH analysis using the custom AmpliSeq™ NGS GWLOH v2 panel (GenomeScan BV, Leiden, The Netherlands), which assesses approximately 1500 SNPs across all autosomes and the X chromosome. These SNP array plots show the median amplicon read count (A–D, top panel) visualizing the quality of the tested sample, the normalized median amplicon read count (A–D, middle panel) visualizing the relative copy number information, and the VAF (A–D, bottom panel) visualizing any chromosomal imbalances. Results are displayed per chromosome (see horizontal numbering in the top row) and separately for p (blue) and q arm (yellow). Figure 1A displays a normal pattern with no CNA, as observed in nodular hyperplasia with oncocytic cell metaplasia (Table 2, case 3). Figure 1B displays the results of the CNA–LOH analysis performed on the histology (tumor cell percentage ≥70%) of an OCA in a male patient (Table 2, case 23), with GH-type CNA with suspected endoreduplication, characterized by clear loss of heterozygosity (A, bottom panel) and chromosomal losses (B, middle panel) of chromosomes 1–4, 6, 8–9, 11, 14, 15, and 20–22. There was some heterogenicity, indicated by the smaller VAF amplitude of chromosomes 15 and 20 as compared to the other affected chromosomes (B, bottom panel). The extreme VAF amplitude (B, bottom panel) of most affected chromosomes is highly suspect of endoreduplication. This neoplasm was diagnosed as molecularly malignant. This case is further illustrated in the Results section. Figure 1C displays the results of the GWLOH panel performed on the histology (tumor cell percentage ≥70%) of an OA in a male patient (Table 2, case 12), with GH-type CNA without endoreduplication, characterized by chromosomal imbalances with less pronounced amplitudes (C, bottom panel) and chromosomal losses (C, middle panel) of affected chromosomes 2, 9, 13, and 22. Limited heterogenicity is possibly observed. This OA was considered molecularly benign. Figure 1D displays an oncocytic cell neoplasm in a female patient (Table 2, case 16). On histopathological assessment, this lesion had a thick capsule and no signs of vascular invasion. Based on a focal interruption of the capsule of equivocal invasive malignant or iatrogenic origin, the neoplasm was considered a minimally invasive OCA. On CNA–LOH analysis, the lesion showed RCI-type CNA, characterized by chromosomal imbalances (D, bottom panel) and relative chromosomal gains (D, middle panel) affecting chromosomes 1, 5, 6, 10, 12, 16, 17, 19, 20, and X. As such, molecularly, this lesion is considered benign. This case is further illustrated in the ‘Results’ section. CNA, copy number alterations; CNA–LOH, copy number alterations and loss of heterozygosity; GH-type, genome-haploidization type; GWLOH, genome-wide loss of heterozygosity; OA, oncocytic thyroid adenoma; OCA, oncocytic cell thyroid carcinoma; RCI-type, reciprocal chromosomal imbalance type; SNP, single-nucleotide polymorphism; VAF, variant allele frequency.

For molecular testing, total nucleic acid (DNA and RNA) was isolated either from formalin-fixed paraffin-embedded (FFPE) histopathology samples using FFPE tissue cores (0.6 mm diameter and variable length), from micro-dissected cytology cell blocks, or from tumor cells scraped off cytology slides (van der Tuin *et al.* 2019*a*, *b*, Cohen *et al.* 2020). NGS was performed on the Ion Torrent GeneStudio™ S5 platform (GenomeScan BV, Leiden, The Netherlands) using custom NGS panels for CNA–LOH analysis, somatic DNA analysis, and gene fusion analysis. CNA–LOH analysis was performed using the custom AmpliSeq™ NGS genome-wide LOH (GWLOH) v2 panel, which assesses LOH and other chromosomal imbalances using 1500 SNPs, evenly distributed across all autosomes and the X chromosome. This analysis has a transit time of only a few days. The results of the GWLOH panel are visualized using SNP array plots (Fig. 1) displaying the median amplicon read count (Fig. 1A, B, C, and D, top panel) visualizing the quality of the tested sample (>2.0 (log_10_(100) = 2)) is considered good quality), the normalized median amplicon read count (middle panel) visualizing the relative copy number information, and the variant allele frequency (VAF) plot (bottom panel) visualizing any chromosomal imbalances including LOH. A VAF of 0.50 indicates heterozygosity; distinct segregation of the SNPs indicates chromosomal imbalance. Somatic mutation analysis was performed using the custom Ampliseq™ Cancer Hotspot v6 panel (Thermo Fisher Scientific) or the custom Ampliseq™ NGS ENDO32 v1 panel (Thermo Fisher Scientific), which respectively assess 87 and 27 (thyroid) cancer-related genes, as previously described (details provided in Supplementary Data, see section on supplementary materials given at the end of this article) (van der Tuin *et al.* 2019*a*, Cohen *et al.* 2020). Any alterations classified as (likely) pathogenic (i.e., class 4 or 5, respectively) were reported (Plon *et al.* 2008). Gene fusion analysis was performed using the Archer® FusionPlex CTL v1 or v2 panel (ArcherDX Inc., Boulder, CO, USA), which respectively assess fusions in 16 and 19 genes, as previously described (Supplementary Data) (van der Tuin *et al.* 2019*a*,*b*, Cohen *et al.* 2020).

### Copy number alteration patterns

**Figure 2:**
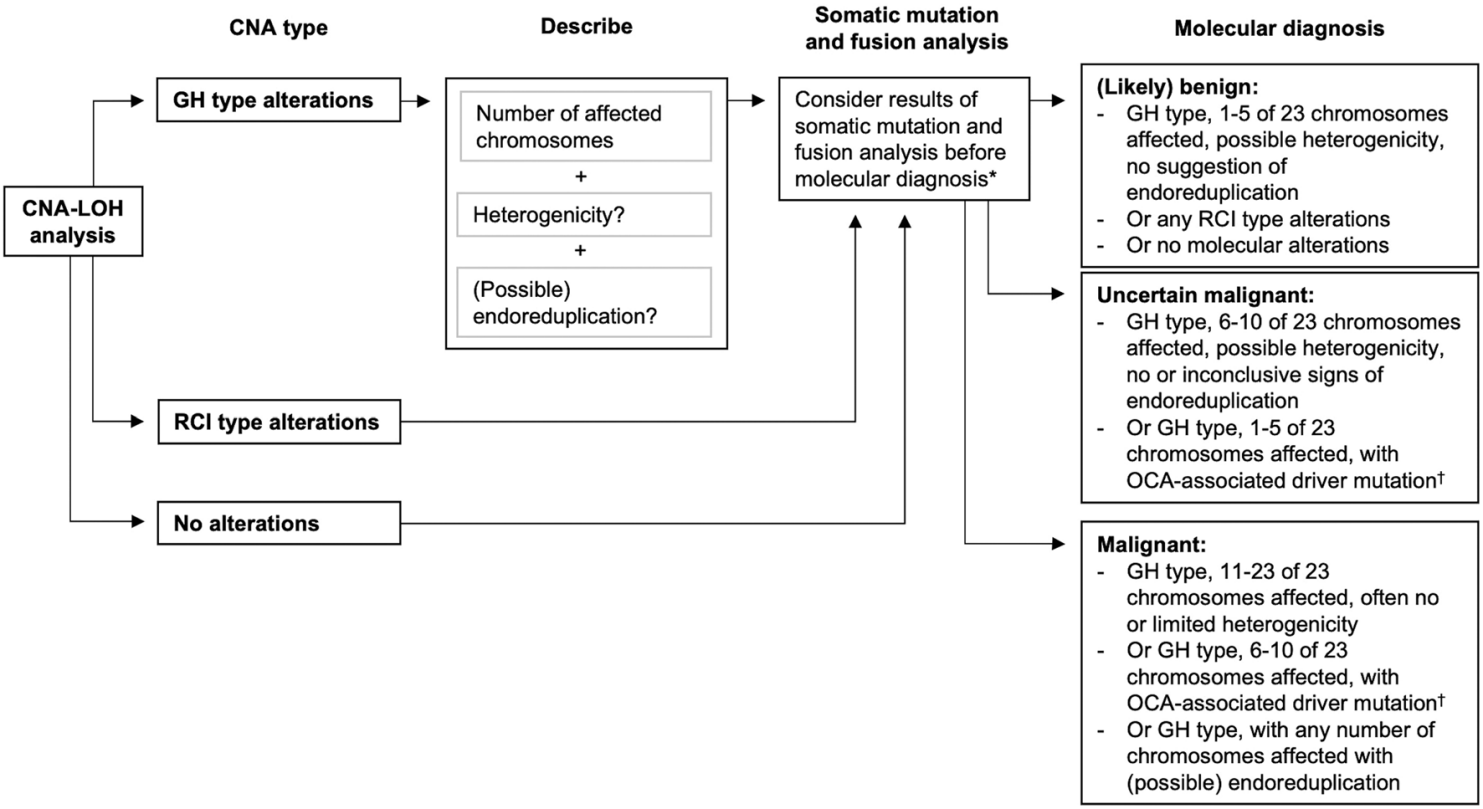
Flowchart for the systematic interpretation of the SNP array plots visualizing the results of CNA–LOH analysis using the GWLOH v2 panel, in order to establish a molecular diagnosis. First, the CNA type is identified as GH-type, RCI-type, or no CNA. GH-type CNA, defined by loss of heterozygosity and chromosomal losses, are further characterized by assessing the number of affected chromosomes, the presence of heterogenicity of the alterations among the affected chromosomes, and the possible presence of endoreduplication. RCI-type CNA are defined by (imbalanced) chromosomal copy number gains. No further characterization of these alterations is needed. Next, the results of somatic mutation and fusion analysis should be considered alongside the results of the CNA–LOH analysis. Finally, the molecular diagnosis is determined as (likely) benign, uncertain malignant, or malignant. *in case a somatic mutation or gene fusion is identified that is uncommon in oncocytic cell neoplasms, whether or not in combination with atypical CNA patterns, reevaluate the presence of *true* oncocytic cells in the sample (also see Box 1) and consider alternative diagnoses that may present with oncocytic cell metaplasia; ^†^including but not limited to TERT promoter or TP53 mutations. CNA, copy number alterations; CNA–LOH, copy number alterations and loss of heterozygosity; GH-type, genome-haploidization type; GWLOH, genome-wide loss of heterozygosity; RCI-type, reciprocal chromosomal imbalance type.

The results of the CNA–LOH analysis using the GWLOH panel were interpreted as described in the flowchart in Fig. 2, identifying the different types of CNA patterns and establishing a molecular diagnosis. First, the CNA type is identified as GH-type, reciprocal chromosomal imbalance (RCI) type, or no CNA. GH-type CNA are defined by LOH and chromosomal losses, as observed by chromosomal imbalances on the VAF plot (Fig. 1B andC, bottom panel) and a *lower* normalized median amplicon read count of the affected chromosomes as compared to the unaffected chromosomes, indicating copy number losses (Fig. 1B and C, middle panel). This CNA pattern requires further characterization by assessing the number of affected chromosomes, any heterogenicity of the alterations, and the possible presence of endoreduplication (Fig. 2). We defined three categories for the number of affected chromosomes: 1–5, 6–10, and 11–23. Heterogenicity is defined as varying VAF amplitudes of the chromosomal imbalances among the affected chromosomes (Fig. 1B, bottom panel) and is associated with benign disease. Endoreduplication is also assessed using the VAF plot amplitudes. GH-type CNA with (suspected) endoreduplication (Fig. 1B) are characterized by an extreme amplitude of the SNP segregation of the LOH-affected chromosomes (Fig. 1B, bottom panel). GH-type CNA without endoreduplication (Fig. 1C) are characterized by other, non-extreme VAF amplitudes of the chromosomal imbalances (Fig. 1C, bottom panel). As the former is associated with OCA, primarily higher stage disease, and the latter is observed in both OA and OCA, it is important to aim to distinguish these two clinically relevant subtypes of the GH-type CNA pattern (Tallini *et al.* 1999, Wada *et al.* 2002, Stankov *et al.* 2004, Corver *et al.* 2012, 2014, 2018, Ganly *et al.* 2018, Gopal *et al.* 2018, Jalaly & Baloch 2020, Doerfler *et al.* 2021).

RCI-type CNA (Fig. 1D) are characterized by (imbalanced) chromosomal copy number gains, as observed by an SNP imbalance on the VAF plot (Fig. 1D, bottom panel) and a *higher* normalized median amplicon read count of the affected chromosomes (Fig. 1D, middle panel), indicating copy number gains. In contrast to GH-type CNA, all chromosomes may be involved. No further characterization of RCI-type CNA are required (Fig. 2). The RCI-type, consistent with genotype AAB, is foremost associated with benign oncocytic cell proliferations (Wada *et al.* 2002, Corver *et al.* 2018). No CNA are observed when the VAF plot indicates heterozygosity (Fig. 1A, bottom panel) and the normalized median amplicon read count is similar across all chromosomes (Fig. 1A, middle panel).

Next, the results of somatic mutation and fusion analysis are considered alongside the results of the CNA–LOH analysis (Fig. 2). When additional driver mutations that are associated with OCA (e.g., TERT promoter, TP53) are observed, a malignant molecular diagnosis is considered more likely.

Finally, the molecular diagnosis is categorized as (likely) benign, uncertain malignant, or malignant. We predefined a (likely) benign molecular diagnosis as limited GH- type CNA, with 1–5 affected chromosomes, possible heterogenicity of the alterations, and no signs of endoreduplication, or any RCI-type alterations, or no molecular alterations on CNA–LOH, somatic mutation, and fusion analysis. An uncertain malignant diagnosis was predefined as GH-type alterations in 6–10 chromosomes or limited GH-type alterations in 1–5 chromosomes in combination with an OCA-associated driver mutation. Heterogenicity of the alterations may be present among the affected chromosomes; no inconclusive signs of endoreduplication are observed. A malignant molecular diagnosis is defined as extensive GH-type CNA affecting 11–23 chromosomes, often with limited or without heterogenicity, or GH-type alterations in 6–10 chromosomes in combination with an OCA-associated driver mutation, or GH-type CNA with any number of affected chromosomes and suspected endoreduplication.

### CNA pattern verification

**Figure 3:**
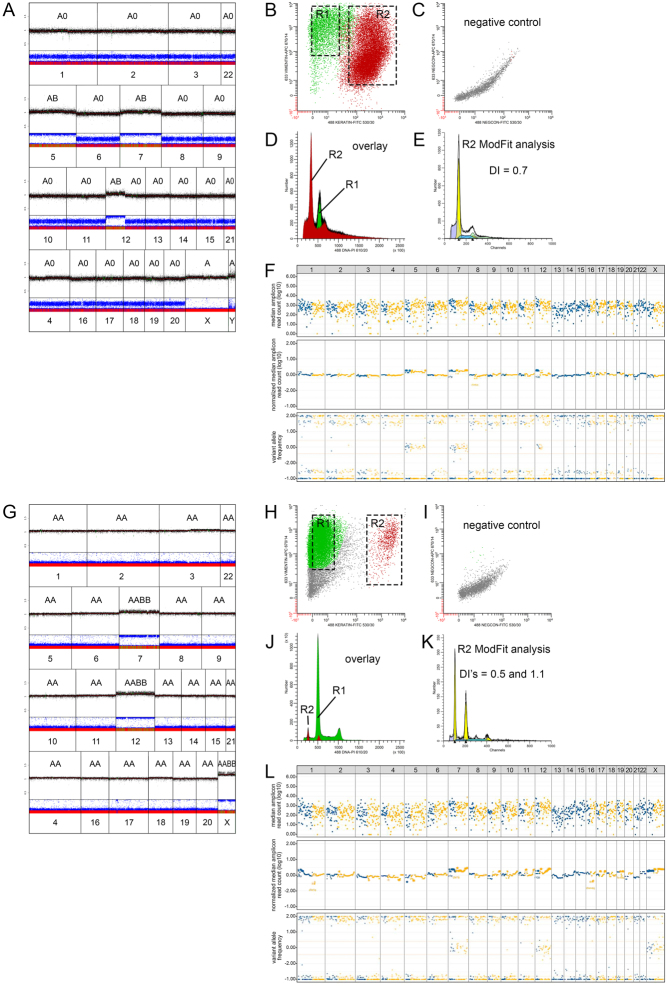
Verification of the CNA patterns observed during CNA–LOH analysis using the GWLOH v2 NGS panel (F and L) in historical cases on which LAIR analysis (A–E and G–K) was previously performed according to the methods as described by Corver *et al.* (Corver *et al.* 2018). One OCA (A–F) and one ATC-H (G–L) are presented here. (A) The SNP array analysis (iCOG and HumanCytoSNP-12, Illumina, Inc., San Diego, CA, USA) of the OCA in a male patient showed homozygosity and chromosomal losses of chromosomes 1–4, 6, 8–11, 12q, and 13–22. (B and H) Keratin (FITC) versus vimentin (APC) fluorescence. Different populations R1 (vimentin positive, keratin negative) and R2 (vimentin positive and keratin positive) can be identified. (C and I) Negative control stained with the secondary reagents and PI only. Note the difference in fluorescence intensities as compared to keratin and vimentin-stained samples (B and H). (D and J) Overlay of population R1 and R2. Note that the major G_0_G_1_-peak of the histogram is painted red and left of the green G_0_G_1_-peak (the DNA-diploid internal reference, minor in D and major in J), indicative of loss of DNA. (E and K) The MFI of the G_0_G1-of R1 was used to accurately calculate the DNA index of the major G_0_G_1_-peak of R2. (E) The DNA histogram of R2, the keratin-positive, vimentin-positive population showed debris for which the ModFit LT algorithm corrects. The DNA index was 0.7. (A–E) Together, these findings indicated the loss of DNA and near-haploidy, corresponding to an A0 genotype. (G) The iCOG SNP array of the ATC-H in a female patient showed homozygosity and chromosomal losses of chromosomes 1–6, 8–11, and 13–21. (K) The DNA histogram of R2, the keratin-positive, vimentin-positive population showed to be bi-modal due to two cycling populations, with DNA indices of 0.5 (i.e., near-haploid tumor fraction) and 1.1 (i.e., fraction with endoreduplication), respectively. (G–K) Together, these findings showed loss of DNA, near-haploidy and endoreduplication of the entire near-haploid population. (F and L) On the GWLOH panel, both cases showed chromosomal losses of the affected chromosomes relative to the unaffected chromosomes (middle panels, normalized median amplicon read count). The VAF of the OCA (F, bottom panel) showed a less pronounced amplitude of the SNP segregation than the ATC-H (L, bottom panel), corresponding to GH-type CNA without (F) and with (L) endoreduplication, respectively. As such, the CNA patterns observed using the GWLOH panel were consistent with the results of the LAIR analysis. The two other historical cases are presented in Supplementary Fig. 1. 488 or 633, laser wavelength used for excitation. 530/30, bandpass filter used to collect FITC fluorescence (green). 670/14, bandpass filter used to collect APC fluorescence (infrared). >610/20, long pass filter used to collect PI fluorescence (deep-red). APC, allophycocyanin. ATC-H, anaplastic thyroid carcinoma with oncocytic changes. CNA, copy number alterations. CNA–LOH, copy number alterations and loss of heterozygosity; FITC, fluorescein isothiocyanate; GH-type, genome haploidization type; GWLOH, genome-wide loss of heterozygosity; OCA, oncocytic thyroid carcinoma; LAIR, lesser-allele intensity-ratio; MFI, median fluorescence intensity; RCI-type, reciprocal chromosomal imbalance type; SNP, single-nucleotide polymorphism; VAF, variant allele frequency.

To verify the GH-type CNA patterns and appurtenant genotypes that are distinguished using the GWLOH v2 panel, CNA–LOH analysis using the GWLOH panel was performed on four representative historical cases (three OCA and one anaplastic thyroid carcinoma with oncocytic changes, not included in the study cohort) in which LAIR analysis was previously performed according to the methods as previously described by Corver *et al.* (Corver *et al.* 2018). These included two cases with GH- type CNA with suspected endoreduplication of the near-haploid genome (i.e., AA genotype) and two cases with GH-type CNA without endoreduplication (i.e., A0 genotype). For each of these historical cases, the findings of the GWLOH panel were consistent with the findings of the LAIR analysis (Fig. 3, Supplementary Data).

**Figure 4:**
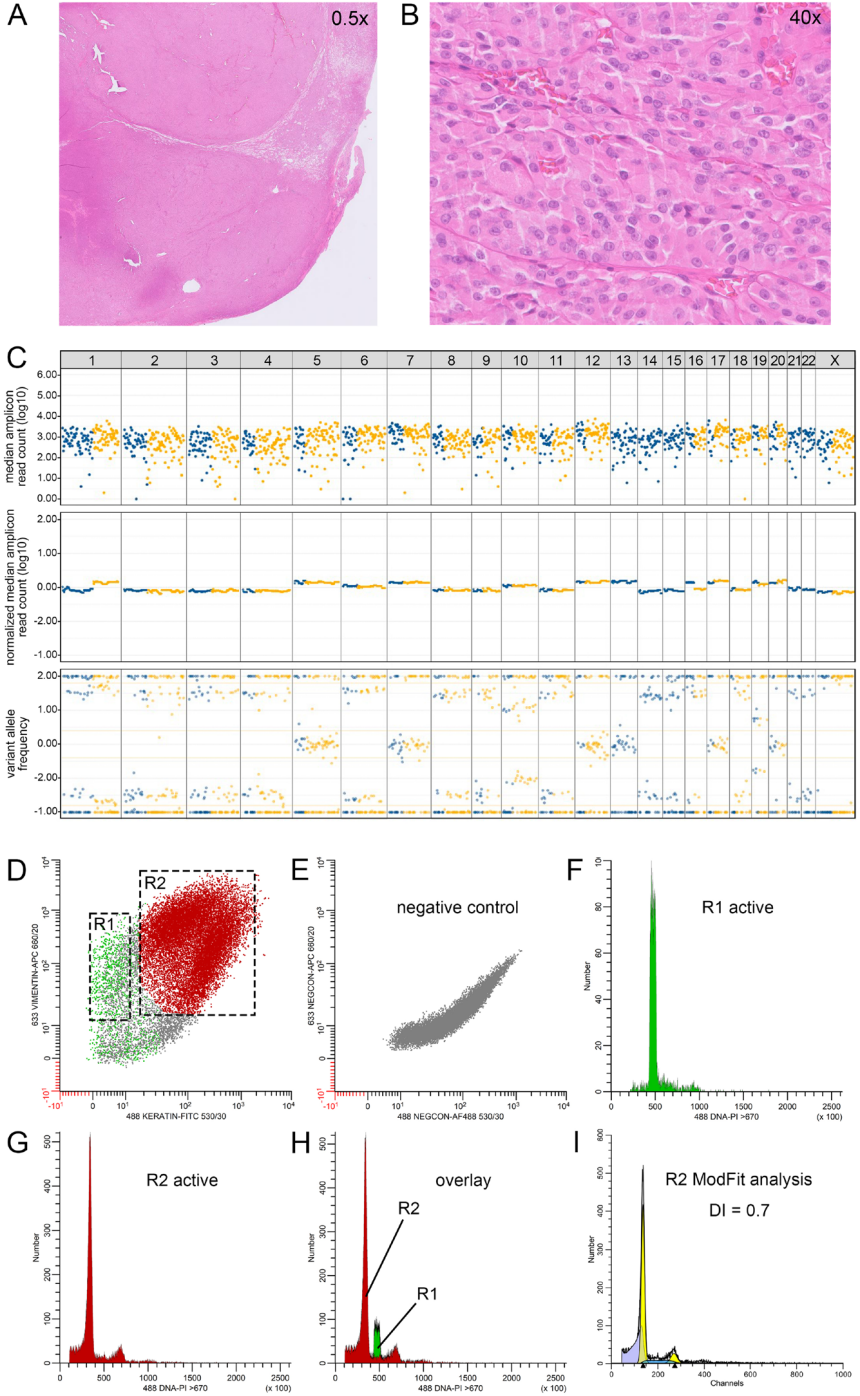
An 85 mm widely invasive oncocytic thyroid carcinoma with GH-type CNA (Table 2, case 29) (A and B). Hematoxylin and eosin-stained sections (A, 0.5×, 2 mm, and B, 40×, 50 μm) from a formalin-fixed, paraffin-embedded specimen of the thyroid tumor. Punches from the same tissue block were used for CNA–LOH analysis using the GWLOH panel and for multiparameter DNA content flow cytometry. (C) Results of the GWLOH panel (tumor cell percentage at least 80%) showed GH-type CNA, with losses of chromosomes 1, 2, 3, 4, 6, 8, 9, 10, 11, 14, 15, 16. 18. 21, and 22, some heterogenicity, and possible endoreduplication. (D–I) Results of the multiparameter DNA content flow cytometry. (D) Keratin (FITC) versus. vimentin (APC) fluorescence. Different populations R1 (vimentin positive, keratin negative) and R2 (vimentin positive and keratin positive) can be observed. (E) Negative control stained with the secondary reagents and PI only. (F) DNA histogram of R1 (green), the vimentin-positive, keratin-negative DNA-diploid (internal reference) stromal population. (G) DNA histogram of R2 (red), the vimentin-positive, keratin-positive population epithelial cell fraction. (H) Overlay of population R1 and R2. Note that the major G_0_G_1_-peak of the histogram is painted red and to the left of the green G_0_G_1_-peak (the DNA-diploid internal reference), clearly indicating loss of DNA and thus likely representing a near-haploid carcinoma population. (I) The MFI of the G_0_G_1_-population of R1 calculated by WinList 3D was linked to ModFit LT allowing accurate calculation of the DNA index of the major G_0_G_1_-peak of R2. The DNA index was 0.7, confirming DNA near-haploidy and the GH-type CNA pattern that was observed using the GWLOH panel. Although endoreduplication was deemed possible on the GWLOH panel, flow cytometry results indicated that no endoreduplication was present (i.e., A0 genotype). On somatic mutation and fusion analysis, no driver mutations were identified. This tumor was diagnosed as molecularly malignant. 488 or 633, laser wavelength used for excitation. 530/30, bandpass filter used to collect FITC fluorescence (green). 660/20, bandpass filter used to collect APC fluorescence (infrared). >670, long pass filter used to collect PI fluorescence (deep-red). APC, allophycocyanin; CNA, copy number alterations; CNA–LOH, copy number alterations and loss of heterozygosity; FITC, fluorescein isothiocyanate; GH-type, genome haploidization type; GWLOH, genome-wide loss of heterozygosity; MFI, median fluorescence intensity.

**Figure 5:**
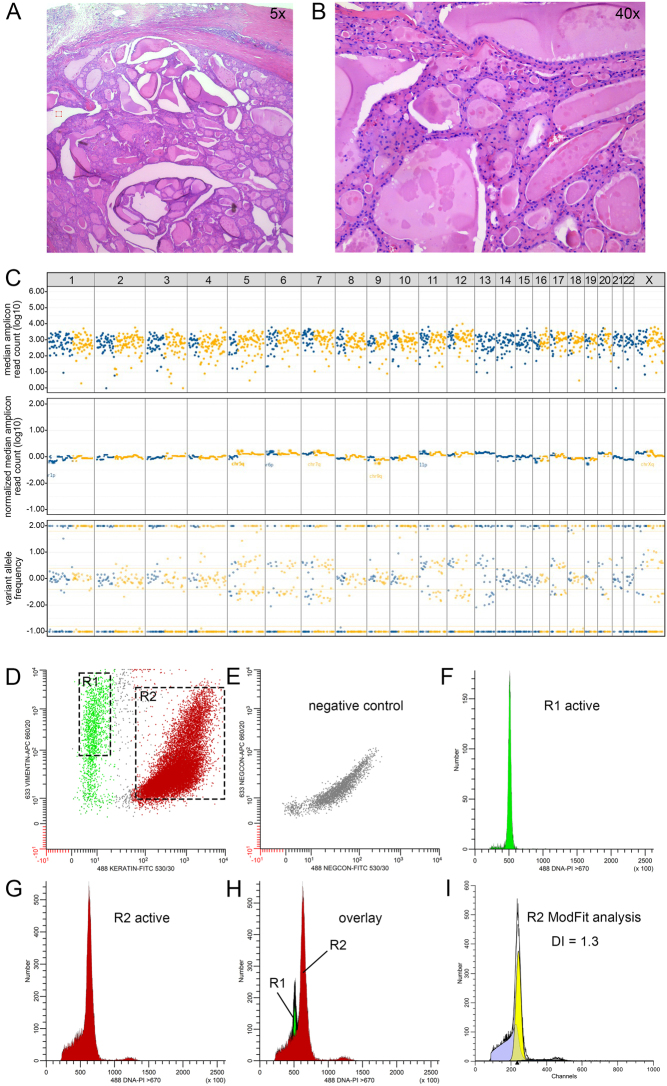
A 58 mm oncocytic thyroid adenoma with RCI-type CNA (Table 2, case 9). (A and B) Hematoxylin and eosin-stained sections (A, 5x, and B, 40×) from a formalin-fixed, paraffin-embedded specimen of the tumor. Punches from the same tissue block were used for CNALOH analysis using the GWLOH panel and for multiparameter DNA content flow cytometry. (C) The results of the GWLOH panel (tumor cell percentage at least 50%) showed chromosomal imbalances of chromosomes 5, 6, 7, 11, 12, 13, 17, 20 and X based on chromosomal copy number gains, corresponding to RCI-type CNA. (D) Keratin (FITC) versus vimentin (APC) fluorescence, with R1 (vimentin positive, keratin- negative) and R2 (vimentin positive and keratin positive) populations. (E) Negative control stained with the secondary reagents and PI only. (F) DNA histogram of R1 (green), the vimentin positive, keratin negative DNA-diploid (internal reference) stromal population. (G) DNA histogram of R2 (red), the vimentin positive, keratin positive population epithelial cell fraction. (H) Overlay of population R1 and R2. Note that the major G_0_G_1_-peak of the histogram is painted red and to the right of the green G_0_G_1_-peak (the DNA-diploid internal reference), indicative for gain of DNA. (I) The MFI of the R1G_0_G_1_-population was used to accurately calculate the DNA index of the major G_0_G_1_-peak of R2. The DNA index was 1.3, indicating gain of DNA and aneuploidy. As such, the results of the multiparameter DNA content flow cytometry are in accordance with the results of the GWLOH panel. On somatic mutation and fusion analysis, no driver mutations were identified. This tumor was considered molecularly benign. 488 or 633, laser wavelength used for excitation. 530/30, bandpass filter used to collect FITC fluorescence (green). 660/20, bandpass filter used to collect APC fluorescence (infrared). >670, long pass filter used to collect PI fluorescence (deep red). APC, allophycocyanin; CNA, copy number alterations; CNA–LOH, copy number alterations and loss of heterozygosity; FITC, fluorescein isothiocyanate; GH-type, genome haploidization type; GWLOH, genome-wide loss of heterozygosity; MFI, median fluorescence intensity; RCI-type, reciprocal chromosomal imbalance type.

In addition, multiparameter DNA flow cytometry was performed according to the methods as described by Corver *et al.* (Corver *et al.* 2005, 2018), with minor modifications, on FFPE material of two cases from the study cohort: one OCA case (Table 2, case 29) with GH-type CNA (Fig. 4) and one OA case (Table 2, case 9) with RCI-type CNA (Fig. 5). In short, 2 mm tissue punches, taken from the same tissue block as used for the GWLOH panel, were transferred to a recipient paraffin block. Sixty-micron sections were taken, deparaffinized, heat-treated, and dissociated using a mixture of collagen I and dispase with the aid of a gentleMACS^TM^ (Miltenyi BioTec, Bergisch Gladbach, Germany). Next, cell suspensions were labeled for keratin (FITC), vimentin (APC), and DNA by propidium iodide (PI). Cells were treated with RNase to improve the resolution of the DNA histograms. Cells were interrogated using a FACSCanto II flow cytometer (BD Biosciences, San Jose, CA, USA) (Corver *et al.* 2005). For the historical cases (Fig. 3, Supplementary Fig. 1), an LSRII flow cytometer (BD Biosciences, San Jose, CA, USA) was used. All multiparameter DNA content flow cytometry data were analyzed using ModFit^TM^ 6.0, remotely controlled by WinList^TM^ 3D 10.0 (Verity Software House, Topsham, ME, USA). The median relative fluorescence of the vimentin-positive, keratin-negative G_0_G_1_-fraction was used as an internal DNA-diploid reference (Corver *et al.* 2005, 2011).

### Statistical analysis

Where appropriate, parametric and nonparametric data were compared using the one-way ANOVA, and Mann–Whitney *U* or Kruskall–Wallis test, respectively. *P* values from the *post hoc* analysis were adjusted by Bonferroni correction for multiple tests. Categorical data were compared using the two-sided Pearson’s chi-squared or Fisher’s exact test, where appropriate. A *P* value of <0.05 was considered statistically significant. All statistical analyses were performed using Statistical Package for Social Sciences (SPSS) Statistics version 27 (IBM Corp., Armonk, NY, USA).

## Results

### Study cohort baseline

Forty-six patients with CNA–LOH analysis using the GWLOH v2 panel on 48 nodules were screened for eligibility. Fourteen (29%) nodules, all with an ultrasound size smaller than 40 mm, were excluded because no surgery was performed, mainly due to the patient’s age and comorbidities. Four (9%) nodules with CNA–LOH analysis on (oncocytic cell) cytology were excluded because subsequent histopathology revealed a non-oncocytic cell lesion. Finally, 29 patients with 30 nodules were included (Table 1), including three (10%) with a histopathological diagnosis of NH-H, 11 (37%) OA, and 16 (53%) OCA including 13 (43%) minimally invasive (mi-OCA) and three (10%) widely invasive OCA (wi-OCA). There were no nondiagnostic CNA–LOH results.

**Table 1:** Baseline characteristics.

	NH-H	OA	OCA	
*n* (patients)	3	10	16	*P*
*n* (nodules)	3	11	16	
Female, *n* (%)	3 (100%)	7 (70%)	8 (50%)	0.21^a,b^
Mean age, years (± SD)^c^	55 ± 17	60 ± 12	54 ± 12	0.53^d,b^
Median nodule size on histopathology, mm (IQR)	35 (15–35)	25 (17–40)	38 (21–50)	0.41^e^
Bethesda classification				0.80^a^
III	–	–	1 (6%)	
IV	3 (100%)	7 (64%)	9 (56%)	
V	–	1 (13%)	2 (13%)	
not available	–	3 (27%)	4 (25%)	
Molecular testing				
on cytology	3 (100%)	3 (27%)	6 (37.5%)	0.07^a^
on histopathology	0 (0%)	8 (73%)	10 (62.5%)	

^a^Pearson’s chi-squared test; ^b^calculated on total number of patients; ^c^mean age at the time of the CNA–LOH analysis; ^d^one-way ANOVA; ^e^Kruskall–Wallis test. OA, oncocytic thyroid adenoma; OCA, oncocytic thyroid carcinoma; IQR, interquartile range; SD, standard deviation; NH-H, nodular hyperplasia with oncocytic cell metaplasia.

### Copy number alterations–loss of heterozygosity analysis

**Figure 6:**
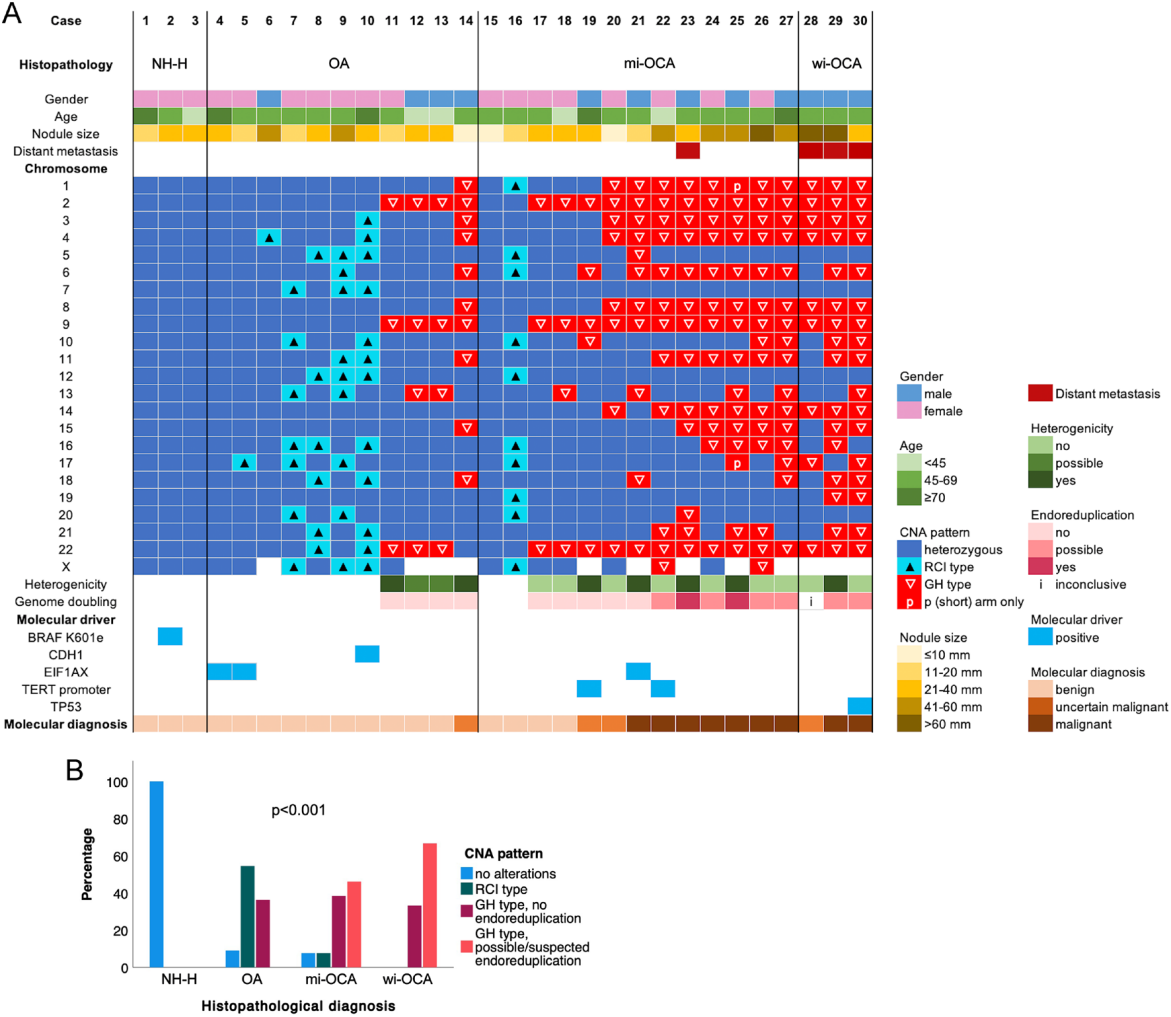
(A) Molecular alterations observed in 30 oncocytic cell nodules based on CNA–LOH analysis using the GWLOH v2 panel, and somatic point mutation and gene fusion analysis. Nodules 12 and 13 are a left- and right-sided nodule in the same patient. Although CNA patterns appear similar in both nodules, further analysis of the SNP profiles demonstrated that the GH-type alterations appeared in different alleles on chromosomes 13 and 22 of the two nodules. The lesions were therefore considered of different clonal origin and both included in the current study. (B) Clustered bar chart showing the statistically significantly different rate of the CNA patterns among the different histopathological diagnoses (*P <* 0.001). CNA, copy number alterations. GH-type, genome haploidization type; GWLOH, genome-wide loss of heterozygosity; mi-OCA, minimally invasive oncocytic thyroid carcinoma; NH-H, nodular hyperplasia with oncocytic cell metaplasia; OA, oncocytic thyroid adenoma; RCI-type, reciprocal chromosomal imbalance type; Wi-OCA, widely invasive oncocytic thyroid carcinoma.

On CNA–LOH analysis using the GWLOH panel, CNA were observed in 25 of 30 (83%) lesions, including 10 of 11 (91%) OA, 12 of 13 (92%) mi-OCA, and 3 of 3 (100%) wi-OCA (Table 2, Fig. 6). The CNA patterns were different between the histopathological subgroups (*P <* 0.001). GH-type CNA was found in 18 nodules, including 4 of 11 (36%) OA, 11 of 13 (85%) mi-OCA, and all wi-OCA. GH-type CNA most frequently included chromosomes 2, 9, and 22, followed by 1, 3, 4, 6, 8, and 14. Chromosomes 7 and 12 were never involved. More chromosomes were affected in OCA (median 12 (IQR 7–16)) than OA (4 (3-9), *P = *0.04) but not in mi-OCA (12 (5-15)) as compared to wi-OCA (17 (8-17), *P = *0.06). Possible endoreduplication was observed in eight lesions, including one patient with American Joint Committee on Cancer stage I, four with stage III, and three with stage IVc disease (*P = *0.009) (Haugen *et al.* 2016). The median number of affected chromosomes in lesions with suspected endoreduplication was higher (15 (IQR 12–17)) than in lesions without (4 (4-9), *P <* 0.001). (Possible) heterogenicity was observed in 4 of 4 (100%) OA and 5 of 14 (36%) OCA with GH–type CNA (*P = *0.08). RCI–type CNA were observed in seven nodules, including 6 of 11 (55%) OA and one equivocal case of mi-OCA (Table 2, case 16, further discussed below). CNA were detected as the only genetic alteration in 12 of 16 (75%) OCAs. Three mi-OCA and one wi-OCA showed an additional molecular driver, including one EIF1AX, two TERT promoters, and one TP53 mutation.

**Table 2:** Clinical, histopathological, and GWLOH data per included nodule (*n* = 30).

no.	Age/Sex	Lesion size (mm)	Histopathology	FU	Status	MD material	Tumor cell %	CNA type	Chromo-somes (*n*)	Hetero-geneity	Endoreduplication	Molecular driver (DNA + RNA)	Molecular diagnosis
1	74F	15	NH-H	5	NED	Cytology	≥90%	No CNA	3	3	3	None	Benign
2	39F	35	NH-H	8	NED	Cytology	≥30%	No CNA	3	3	3	None	Benign
3	54F	40	NH-H	13	NED	Cytology	≥30%	No CNA	3	3	3	BRAF non-v600E, c.1803A>T	Benign
4	76F	27	OA	0	NED	Histology	≥50%	No CNA	3	3	3	EIF1AX, c.338-1G>C	Benign
5	66F	19	OA	7	NED	Cytology	≥90%	RCI-type	3	3	3	EIF1AX, c.338-2A>T	Benign
6	55M	49	OA	5	NED	Histology	≥80%	RCI-type	3	3	3	None	Benign
7	48F	17	OA	1	NED	Cytology	≥30%	RCI-type	3	3	3	None	Benign
8	62F	40	OA	2	NED	Cytology	≥30%	RCI-type	3	3	3	None	Benign
9	49F	58	OA	?	?	Histology	≥50%	RCI-type	3	3	3	None	Benign
10	76F	25	OA	?	?	Histology	≥70%	RCI-type	3	3	3	CDH1, c.220C>T	Benign
11	55F	14	OA	6	NED	Histology	≥50%	GH-type	3 / 23	Yes	No	None	Benign
12^a^3	44M	25	OA	?	?	Histology	≥70%	GH-type	4 / 23	Possible	No	None	Benign
13^a^3	44M	25	OA	?	?	Histology	≥70%	GH-type	4 / 23	Possible	No	None	Benign
14	69M	3	OA	?	?	Histology	≥70%	GH-type	10 / 23	Yes	No	None	Uncertain malignant
15	67F	10	mi-OCA, pT1aN0M0	36	NED	Histology	≥50%	no CNA	3	3	3	None	Benign
16	63F	20	mi-OCA, pT1bN0M0	38	NED	Histology	≥80%	RCI-type	3	3	3	None	Benign
17	50F	22	mi-OCA, pT2N0M0	?	?	Histology	≥80%	GH-type	3 / 23	No	No	None	Benign
18	33F	27	mi-OCA, pT2N0M0	22	NED	Histology	≥60%	GH-type	4 / 23	No	No	None	Benign
19	78M	36	mi-OCA, pT2N0M0	?	?	Histology	≥80%	GH-type	5 / 23	Yes	No	TERT, c.-124C>T	Uncertain malignant
20	49V	6	mi-OCA, pT1aN0M0	4	NED	Cytology	≥30%	GH-type	8 / 23	No	No	None	Uncertain malignant
21	46M	14	mi-OCA, pT1bN0M0	6	NED	Histology	≥80%	GH-type	11 / 23	Yes	No	EIF1AX, c.338-2A>G	Malignant
22	39F	50	mi-OCA, pT3N0M0	17	NED	Cytology	≥50%	GH-type	12 / 23	No	Possible	TERT, c.-124C>T	Malignant
23	57M	27	mi-OCA, pT2N1bM1, with pulmonary and renal metastases	161	AWD	Histology	≥70%	GH-type	13 / 23	Yes, limited	Yes	None	Malignant
24	51F	44	mi-OCA, pT3N0M0	7	AWD	Cytology	≥50%	GH-type	12 / 23	No	Possible	none	Malignant
25	54M	50	mi-OCA, pT3N0M0	11	AWD	Histology	≥60%	GH-type	15 / 23	Yes, limited	Yes	None	Malignant
26	54F	78	mi-OCA, pT3N0M0	1	AWD	Histology	≥80%	GH-type	15 / 23	No	Possible	None	Malignant
27	73M	48	mi-OCA, pT3N0M0	?	?	Core biopsy	≥70%	GH-type	17 / 23	No	Possible	None	Malignant
28	46M	80	wi-OCA, pT3N0M1, with extensive vascular invasion, pulmonary and osseous metastases	20	AWD	Cytology	≥30%	GH-type	8 / 23	No	Inconclusive	None	Uncertain malignant
29	61M	85	wi-OCA, pT3N1bM1, with extensive lnn, pulmonary and osseous metastases	17	AWD	Cytology	≥80%	GH-type	17 / 23	Yes	Possible	None	Malignant
30	49M	40	wi-OCA, pT3N1bM1, with extensive lnn, pulmonary, and osseous metastases	20	DOD	Histology	≥50%	GH-type	18 / 23	No	Possible	TP53, c.1010_1011dupGC	Malignant

^a^Nodules 12 and 13 are a left- and right-sided nodule in the same patient. Although CNA patterns appear similar in both nodules, further analysis of the SNP profiles demonstrated that the GH-type alterations appeared in different alleles on chromosomes 13 and 22 of the two nodules. The lesions were therefore considered of different clonal origin and both included in the current study. ‘?’ follow-up status of patient is unknown. AWD, alive with disease; CHS, cancer hotspot; CNA, copy number alterations; DOD, died of disease-related causes; F, female; FU, follow-up duration in months, measured from date of primary thyroid surgery until date of latest follow-up visit; GH-type, genome haploidization type; M, male; mi-OCA, minimally invasive oncocytic thyroid carcinoma; NED, no evidence of disease; NH-H, nodular hyperplasia with oncocytic cell metaplasia; OA, oncocytic thyroid adenoma; RCI-type, reciprocal chromosomal imbalance type; wi-OCA, widely invasive oncocytic thyroid carcinoma.

Morphological and molecular diagnoses were discordant case 14, 15, 16 (illustrated below), 17, 18, and 23 (illustrated below) (Table 2, Fig. 6).

The histopathological nodule size was similar in benign and malignant nodules (*P = *0.19), but significantly different between CNA patterns (*P = *0.02). *Post hoc* analysis showed that this difference was based on a larger size in nodules with GH-type CNA with suspected endoreduplication than in GH-type nodules without endoreduplication (*P = *0.01). Nodule size was similar among other CNA patterns.

### Clinical cases

#### Case 23

**Figure 7:**
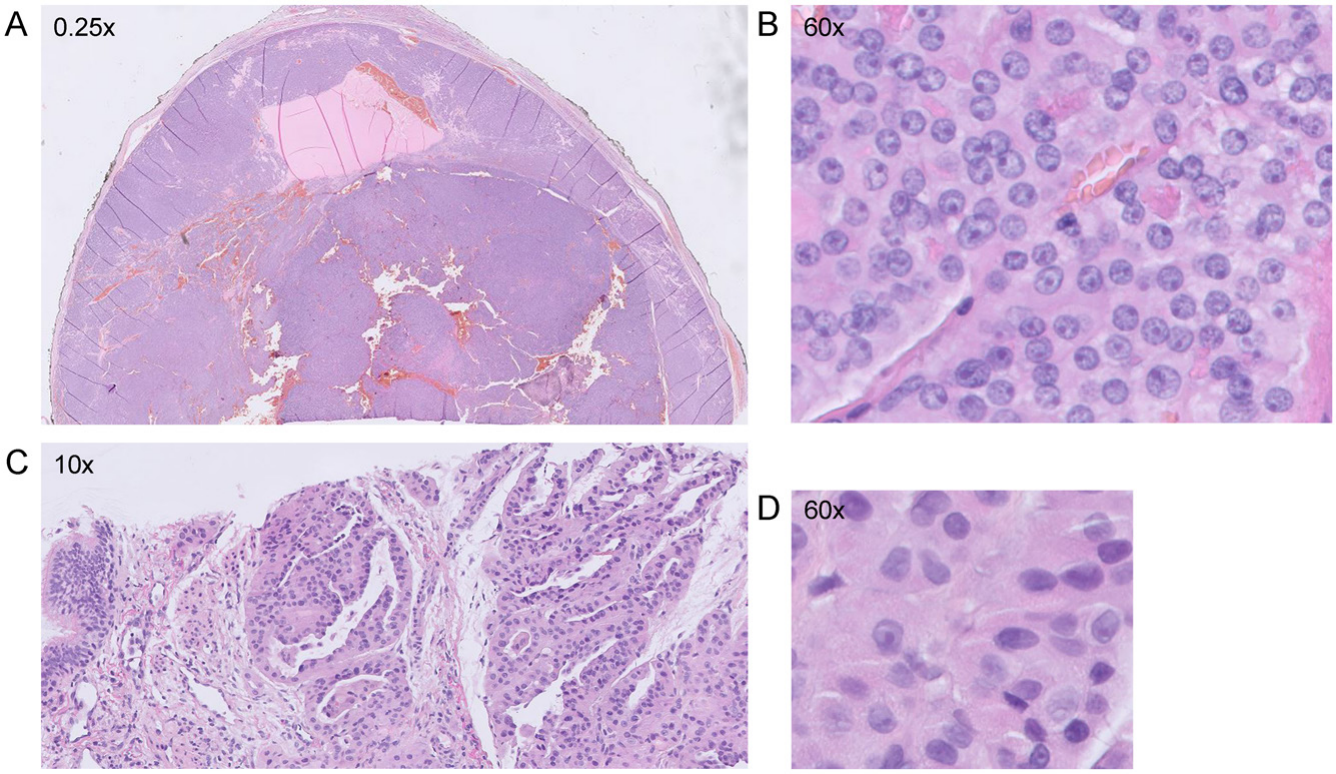
Case 23 (Table 2). (A, B) Hematoxylin and eosin-stained sections of the primary thyroid tumor, a 27-mm oncocytic cell neoplasm (A, 0.25× and B, 60×) showing large nucleoli, (pseudo-)papillary architecture without papillary nuclear features, and a thin capsule with no signs of capsular or vascular invasion. It was initially morphologically diagnosed as a oncocytic thyroid adenoma and treated as such. Twelve years later, the patient presented with a pulmonary metastasis of this neoplasm (Hematoxylin and eosin-stained sections C, 10× and D, 60×). Results of the CNA–LOH analysis of this patient are presented in Fig. 1B.

A 57-year-old male patient, who was referred to our tertiary care center with lymphatic, pulmonary, and bilateral renal metastases of an OCA (Table 2). Twelve years earlier, he had undergone a diagnostic hemithyroidectomy for a right-sided thyroid nodule. At the time, it was morphologically diagnosed as an OA and no further treatment was considered necessary. A recent review of the histopathology slides supported the original *morphological* diagnosis, showing a 27-mm OCN with large nucleoli, (pseudo-)papillary architecture without papillary nuclear features, and a thin capsule, without signs of capsular or vascular invasion (Fig. 7A and B). A biopsy of the pulmonary metastasis showed similar histopathological characteristics (Fig. 7C and D), indicating that, in retrospect, the known OCN should likely be considered an OCA (T2N1bM1). CNA–LOH analysis using the GWLOH panel was performed on the biopsy of the pulmonary metastasis and on preserved FFPE material of the primary tumor. The primary tumor showed extensive GH-type CNA with high suspicion of endoreduplication, involving chromosomes 1–4, 6, 8, 9, 11, 14, 15, 20–22 (Fig. 1B), also denoting the malignant nature of the lesion. No point mutations or gene fusions were found. A similar CNA pattern was observed in the pulmonary metastasis, confirming its origin. No contralateral thyroid tumor was present. The patient recently underwent a completion thyroidectomy and adjuvant radioiodine therapy.

#### Case 16

A 63-year-old female patient, who presented with a palpable, left-sided thyroid nodule from which Bethesda IV cytology was obtained (Table 2). She underwent a diagnostic hemithyroidectomy, revealing a 20-mm OCN with a thick capsule and without signs of vascular invasion. On histopathological assessment, the tumor capsule showed a focal interruption of equivocal invasive malignant or iatrogenic origin. Immunohistochemistry showed no loss of PTEN protein expression and wild-type p53 expression. Based on the possible capsular invasion, the neoplasm was initially considered a mi-OCA (T1bN0Mx). CNA–LOH analysis showed RCI-type CNA of chromosomes 1, 5, 6, 10, 12, 16, 17, 19, 20, and X (Fig. 1D). No somatic mutations or gene fusions were found using the Ampliseq™ Cancer Hotspot v6 and the Archer® FusionPlex CTL v2 panels. As such, this OCN was molecularly diagnosed as (likely) benign. The patient and her endocrinologist agreed to a follow-up regimen, refraining from completion thyroidectomy. Thus, the patient underwent a dedicated neck ultrasound every 6 months, to date revealing no abnormalities in 38 months of follow-up. The patient currently remains in follow-up.

## Discussion

In the current study, we described the CNA patterns that were observed on CNA–LOH analysis in benign and malignant OCN using a limited, 1500 SNP GWLOH NGS panel. With this method that is feasible for daily clinical practice, we are able to distinguish four CNA patterns and establish a molecular diagnosis with an increasing risk of malignancy. The assessment of the CNA patterns in a patient cohort confirmed that GH-type CNA is found primarily in OCA but to a lesser extent also in OA. GH-type alterations with possible endoreduplication were reserved for OCA, mi-OCA as well as wi-OCA, and mainly those with more extensive chromosomal losses. These results are in accordance with previous studies that reported on the early and late events in near-whole genome haploidization (Corver *et al.* 2011, 2012, 2014, 2018, Ganly *et al.* 2018, Gopal *et al.* 2018, Jalaly & Baloch 2020, Doerfler *et al.* 2021). Only the loss of chromosome 18 was infrequently observed in the current study, in contrast to observations by Corver *et al.* and Ganly *et al.* (Corver *et al.* 2018, Ganly *et al.* 2018, Gopal *et al.* 2018). RCI-type CNA, which have less frequently been described in literature, were foremost associated with benign disease in the current study (Wada *et al.* 2002, Corver *et al.* 2018). Point mutations were rarely observed and gene fusions were not found in our cohort, endorsing that CNA are likely the main molecular drivers in OCN.

CNA–LOH analysis may be of great added value in the (preoperative) diagnosis and risk stratification of OCN in clinical practice, aiding multidisciplinary patient management decisions. Case 23 illustrated that CNA–LOH analysis can be pivotal in identifying aggressive biological potential in OCN, especially if morphological histopathological features of malignancy are lacking. Although infrequently reported, metastases of OCN that were initially morphologically diagnosed as OA are notorious (Grant *et al.* 1988, Boronat *et al.* 2013). In hindsight, based on the molecular profile of the original tumor, treating the original lesion of case 23 as an OCA would have been justified. Unfortunately, these molecular techniques were not available yet at the time of the original diagnosis. Case 16 illustrated that CNA–LOH analysis may also contribute to the risk reduction and avoid possible overtreatment of OCN with an equivocal histopathological diagnosis of OCA. Based on the molecularly benign CNA pattern that was observed in this case, indolent biological behavior or even a benign nature of the neoplasm may be expected (Corver *et al.* 2018).

Following the results of the current and previous studies, initial total thyroidectomy could be considered instead of diagnostic hemithyroidectomy for oncocytic cell lesions with (extensive) GH-type alterations and (suspected) endoreduplication (i.e., a malignant molecular diagnosis) (Corver *et al.* 2012, 2014, Ganly *et al.* 2018, Gopal *et al.* 2018, Jalaly & Baloch 2020, Doerfler *et al.* 2021). In less extensive GH-type alterations without (suspected) endoreduplication (i.e., an uncertain malignant molecular diagnosis), diagnostic hemithyroidectomy is recommended to obtain a definitive diagnosis. When RCI-type CNA are observed, hemithyroidectomy should also be considered. Although associated with biologically benign disease in the current study, evidence regarding RCI-type CNA is still limited and future studies are needed to confirm our observations (Wada *et al.* 2002, Corver *et al.* 2018).

Finally, the absence of CNA alone does not exclude malignancy and does not justify withholding diagnostic hemithyroidectomy for a Bethesda III/IV oncocytic cell nodule. Instead, the results of the CNA–LOH analysis are best interpreted in combination with somatic mutation and fusion analysis results (Fig. 2). Various point mutations have additionally been described in OCA, infrequently in malignancies lacking the typical GH-type CNA patterns. These include DAXX, NF1, ARHGAP35, MADCAM1, ATXN1, UBXN11, TSC1/2, and CDKN1A mutations, mutations characteristic of FTC including RAS, PIK3CA, and PTEN mutations, and those that are characteristic of poorly differentiated and anaplastic thyroid carcinoma including TERT promoter, PIK3CA, PTEN, EIF1AX, ATM, and TP53 (Ganly *et al.* 2013, 2018, Corver *et al.* 2018, Jalaly & Baloch 2020, Kumari *et al.* 2020, Santana *et al.* 2020, Doerfler *et al.* 2021). As in other types of thyroid carcinoma, TERT promoter mutations in OCA are associated with more aggressive tumor behavior, distant metastasis, and tumor dedifferentiation including radioiodine refractory disease (Ganly *et al.* 2018). The somatic mutation and gene fusion NGS panels that were used in the current study included the most important but not all of the OCA-appurtenant molecular alterations that were previously described in the literature. Yet, when no CNA and no somatic mutations or gene fusions are observed, a wait-and-see strategy with active surveillance of the nodule appears oncologically safe and should be considered.

During CNA–LOH analysis using the GWLOH panel, a number of additional considerations are crucial (Box 1). First, to establish the diagnostic value of the CNA–LOH analysis, ascertaining the presence of true oncocytic cells as opposed to oncocytic cell metaplasia is key (WHO Classification of Tumours Editorial Board 2022). A careful morphological assessment (including immunohistochemistry) by a dedicated thyroid pathologist may accurately distinguish most true oncocytic cell lesions from other neoplastic or non-neoplastic disorders that present with oncocytic changes, such as oncocytic papillary thyroid carcinoma, oncocytic medullary thyroid carcinoma, or parathyroid proliferations (Asa & Mete 2021). Such neoplasms also show different genetic alterations (Doerfler *et al.* 2021). The observation of an atypical CNA pattern or a somatic mutation or gene fusion that is uncommon in OCN warrants the critical re-evaluation of the cell type and (non-)oncocytic cell origin of the tumor, including consideration of alternative diagnoses (Lloyd *et al.* 2017).

**Box 1** Consideration points for CNA–LOH analysis in daily clinical practice.

- All assessments should be performed by an experienced thyroid pathologist.
- Confirm the presence of true oncocytic cells in the cytological or histopathological sample by microscopic assessment (Lloyd *et al.* 2017).
- Consider the tumor cell percentage of the tested sample. In case of a suboptimal tumor cell percentage, i.e., 30–50% in the case of testing on cytology, assessing the possible presence of endoreduplication may be more difficult.
- Four main CNA patterns are distinguished:
- GH-type alterations with (suspected) endoreduplication, consistent with genotype AA or a multiple thereof in the affected chromosomes, associated with OCA, progression of disease and worse prognosis; initial total thyroidectomy may be considered instead of diagnostic hemithyroidectomy depended on the clinical patient context.
- GH-type alterations without (suspected) endoreduplication, consistent with genotype A0 in the affected chromosomes. Observed in both OA and OCA, the number of affected chromosomes and presence of heterogenicity define the molecular diagnosis (Fig. 2); diagnostic hemithyroidectomy is recommended to obtain a definitive diagnosis.
- RCI-type with chromosomal copy number gains, genotype AAB, foremost associated with benign, biologically indolent disease. Hemithyroidectomy may be considered.
- No CNA, normal heterozygous pattern.
- The presence of widespread GH-type alterations with suspected endoreduplication likely indicates a biologically more aggressive tumor, even in OCN without signs of capsular or vascular invasion (i.e., morphological OA). (Re-)consideration of a malignant molecular diagnosis is warranted.
- The results of the CNA–LOH analysis should be interpreted alongside the results of somatic mutation and fusion analysis. No CNA are found in nodular hyperplasia with oncocytic cell metaplasia and part of the OA. The absence of CNA alone does not exclude malignancy and does not justify withholding diagnostic surgery for a Bethesda III or IV nodule with cytology suspicious for OCN. If no CNA and no somatic mutations or fusions are detected, however, withholding diagnostic surgery may be considered oncologically safe.
- The results of the CNA–LOH analysis should be interpreted in context of other clinicopathological characteristics, including nodule size.

Next, the tumor cell percentage of the tested tissue sample should always be taken into consideration. Whereas the tumor cell percentage of OCN is mostly 70–80% or higher for histopathological samples, often ensuring clear amplitudes in the SNP array plots, the tumor cell percentage of cytology samples can be limited to 30–50%, resulting in smaller VAF amplitudes and/or more scattered SNP plots. In the latter cases, endoreduplication may present with less extreme amplitudes and may even go unnoticed in the assessment. As such, the GWLOH panel can indicate but not always exclude endoreduplication, and – dependent on the quality of the tissue sample – sometimes no decisive answer regarding the presence of endoreduplication may be obtained. Other techniques such as flow cytometry and LAIR analysis are more reliable for this purpose, but these are not fit for daily clinical application (Corver *et al.* 2008, 2012). Finally, although recognizing endoreduplication may seem critical due to its association with an unfavorable prognosis and metastatic disease, it is important to realize that metastasis has occasionally also been described for tumors without genome doubling (Corver *et al.* 2012, 2014, 2018, Ganly *et al.* 2018, Gopal *et al.* 2018, Jalaly & Baloch 2020, Doerfler *et al.* 2021).

Finally, CNA–LOH analysis results should be carefully interpreted in the context of other clinicopathological characteristics, including nodule size, for example. Larger nodule size (>4 cm, in particular) has previously been associated with a higher risk of malignancy in OCN and worse prognosis in OCA (Lopez-Penabad *et al.* 2003, Santana *et al.* 2019, Doerfler *et al.* 2021). Although the current study found no statistically significant difference in nodule size between benign and malignant lesions, nodules with GH-type CNA and possible endoreduplication were significantly larger than GH-type nodules without endoreduplication.

CNA–LOH analysis may resolve important bottlenecks in the preoperative differentiation of OCN. Besides their unique molecular alterations, OCN oftentimes also show atypical results and lower diagnostic accuracy on other preoperatively applied diagnostics, including ultrasound and positron emission tomography/computed tomography (PET/CT) using 2-[^18^F]fluoro-2-deoxy-d-glucose (FDG) (de Koster *et al.* 2018, 2022, Slowinska-Klencka *et al.* 2020). FDG-PET/CT visualizes (increased) metabolic activity in tissues and is successfully applied for the diagnosis, staging and monitoring of many types of cancers (Boellaard *et al.* 2015). A visually negative FDG-PET/CT accurately differentiates between benign and malignant cytologically indeterminate thyroid nodules. This, however, does not apply to nodules with oncocytic cell cytology, which are almost exclusively strongly FDG positive, likely related to their abundance of mitochondria (de Koster *et al.* 2022).

Due to the increased use of imaging techniques for indications unrelated to the thyroid, thyroid nodules are detected in up to 65% of the general population (Durante *et al.* 2018, Bernet & Chindris 2021). This includes FDG-positive thyroid incidentalomas, which are found in approximately 2% of FDG-PET/CT scans with an approximate 31% malignancy rate (de Leijer *et al.* 2021). The exact number of oncocytic cell lesions among these incidentalomas is unknown but may be substantial due to the pronounced FDG-positivity in these nodules (Pathak *et al.* 2016, de Koster *et al.* 2022). In our screened patient population, 11 of 48 (23%) presented with a PET/CT thyroid incidentaloma. Six of 11 (55%) patients did not undergo surgery, most frequently due to (oncological) comorbidities that were the indication for the FDG-PET/CT. In patients with considerable comorbidities, molecular testing including CNA–LOH analysis may aid the considerations and risks of withholding surgery.

The main limitation of our study is its retrospective design, potentially causing bias. Selection bias may have resulted in a nonrepresentative patient cohort if CNA–LOH analysis and/or thyroid surgery were only selectively performed. Moreover, some of the included patients were consultations from community hospitals, specifically referred to our hospital for CNA–LOH analysis. In addition, the relatively small sample size of our cohort limited further statistical analysis of the observed CNA patterns in relation to clinical and histopathological characteristics. The current study was not designed to assess the diagnostic performance of the GWLOH panel. Larger, prospective validation studies are desired to explicate the CNA-appurtenant risk stratification of OCN and assess (preoperative) diagnostic accuracy parameters of CNA–LOH analysis, including differences in test performance between cytological and histopathological tissue specimens. Such cohorts could also include a wider range of neoplastic or non-neoplastic thyroid and parathyroid disorders that present with oncocytic changes to confirm the absence of OCN-characteristic CNA in these diagnoses. Validation studies from our study group are currently in progress, including molecular diagnostics of the prospective *EfFECTS* trial cohort (ClinicalTrials.gov: NCT02208544) (de Koster *et al.* 2022) and a separate prospective trial on preoperative risk stratification of cytologically indeterminate thyroid nodules using molecular diagnostics. Both include non-oncocytic and oncocytic nodules.

In addition, it currently remains unknown whether more accurate differentiation and risk stratification of OCN using molecular diagnostics, including consequent treatment decisions, finally improves the prognosis of OCA. This requires data that is not yet available, i.e., from large cohort studies with molecular diagnostics and follow-up extending over several decades. Finally, cost-effectiveness should be taken into consideration and formal cost-utility studies should be performed in the future. The joint costs of our molecular panels are approximately €1350 ($1479; $1 = €0.91 on May 2, 2023) per patient with an OCN.

In conclusion, the results of this study demonstrate that CNA–LOH analysis using a limited, 1500 SNP NGS panel is a feasible method for CNA–LOH analysis in OCN in everyday clinical practice. The results of this study, including a full description of the CNA patterns that may be distinguished and considerations for their structured interpretation to establish a molecular diagnosis, may aid the widespread application of CNA–LOH analysis for the preoperative as well as postoperative diagnosis and risk stratification of oncocytic cell lesions.

## Supplementary Materials

Supplementary Material

## Declaration of interest

The authors have no conflicts of interest to declare that are relevant to the content of this article.

## Funding

This research did not receive any specific grant from any funding agency in the public, commercial, or not-for-profit sector.

## Author contribution statement

HM and TW conceptualized the study. HM was the project leader. EK prepared the dataset for analysis, drafted the manuscript, and prepared the tables and figures. EK, WC, and HM verified the data and performed the statistical analysis. All authors contributed to data acquisition and the interpretation of the data and critically reviewed this manuscript. All authors had full access to all the data in the study and approved the manuscript before submission. HM had final responsibility for the decision to submit for publication.
