# Supplementary material for: A clinically applicable molecular classification of oncocytic cell thyroid nodules
Source: Endocr Relat Cancer. 2023 Aug 3;30(9):e230047. doi: 10.1530/ERC-23-0047 (PMC10448578; doi:10.1530/ERC-23-0047)
Supplement: Supplementary Material [file supplementary_material.pdf]

A clinically applicable molecular classification of oncocytic cell thyroid nodules

Elizabeth J. de Koster, Willem E. Corver, Lioe-Fee de Geus-Oei, Wim J.G. Oyen, Dina Ruano, Abbey Schepers, Marieke Snel, Tom van Wezel, Dennis Vriens, Hans Morreau

Supplementary Data

NGS Somatic mutation analysis

The NGS somatic mutation analysis was performed by GenomeScan BV, on the Ion Torrent GeneStudio™ S5 platform (GenomeScan BV, Leiden, The Netherlands), using the custom Ampliseq™ Cancer Hotspot v6 panel or the custom Ampliseq™ NGS ENDO32 v1 panel (Thermo Fisher Scientific, Waltham, MA, USA), according to the methods as previously described (Aydemirli, *et al.* 2021; Cohen, *et al.* 2020; Sibinga Mulder, *et al.* 2017; van der Tuin, *et al.* 2019a).

The custom Ampliseq™ Cancer Hotspot v6 panel analyses the following genes: ABL1, AKT1, ALK, APC, ARAF, ATM, BAP1 (exon 3-17 partial), BRAF, CARD11, CD79A, CD79B, CDC73, CDH1, CDK4, CDKN2A, CIC, CSF1R, CTNNB1, CTNNB1, DDR, DICER, EGFR, EIF1AX, ERBB2, ERBB3, ERBB4, ERCC2, ERCC2, EZH2, FBXW7, FGFR1, FGFR2, FGFR3, FLT3, FOXL2, GNA11, GNAQ, GNAS, H3F3A, H3F3B, HNF1A, HRAS, IDH1, IDH2, JAK2, JAK3, KDR, KIT, KRAS, MAP2K1, MAP2K2, MAP2K4, MAP3K1, MDM2, MED12, MET, MLH1, MPL, MUTYH, MYC, MYD88, MyoD1, NKX2-1, NOTCH1, NTRK1, NPM1, NRAS, PDGFRA, PDGFRB, PIK3CA, POLD1, POLE, PTEN, PTK2, PTPN11, RB1, RET, SMAD4, SMARCB1, SMO, SRC, STK11, TERT-promoter, TP53 (exon 2-11 partial), and VHL.

The Custom Ampliseq™ NGS ENDO32 v1 panel analyses the coding exons of the following genes: ATP1A1, ATP2B3, ARMC5, CACNA1D, KCNJ5, NF1, TSC1, CDKN1A, CDKN1B, CDKN1C, CDKN2A, CDKN2B, CDKN2C, CDKN2D, CDC73, MEN1, TP53. It also analyses the hotspots in the following genes: BRAF, DICER1, EIF1AX, HRAS, KRAS, NRAS, PIK3CA, PTEN, RET and the TERT-promoter.

NGS fusion analysis

The NGS fusion analysis was performed by GenomeScan BV on the Ion Torrent GeneStudio™ S5 platform (GenomeScan BV, Leiden, The Netherlands), using the Archer FusionPlex CTL Panel v1 or v2 (ArcherDX Inc., Boulder, CO, USA), according to the methods as previously described (Aydemirli, *et al.* 2021; Cohen, *et al.* 2020; van der Tuin, *et al.* 2019a; van der Tuin, *et al.* 2019b). It uses both

RNA and DNA. Version 1 can detect fusions with the following target genes: ALK, AXL, CCND1, FGFR1, FGFR2, FGFR3, MET, NRG1, NTRK1, NTRK2, NTRK3, PPARG, RAF1, RET, ROS1, and THADA. It can also detect hotspot mutations in BRAF, EGFR, HRAS, KRAS, and NRAS.

Version 2 detects fusions with the following target genes: ALK, AXL, BRAF, CCND1, FGFR1, FGFR2, FGFR3,GLIS1, GLIS3, MET, NRG1, NTRK1, NTRK2, NTRK3, PPARG, RAF1, RET, ROS1, TERT, and THADA. In addition, it detects hotspot mutations in the following genes: AKT1, ALK, BRAF, CTNNB1, DDR2, DICER1, EGFR, EIF1AX, ERBB2, FGFR1, GNAS, HRAS, IDH1, IDH2, KRAS, MAP2K1, NRAS, PIK3CA, RET, and ROS1.

Supplementary Figure 1.

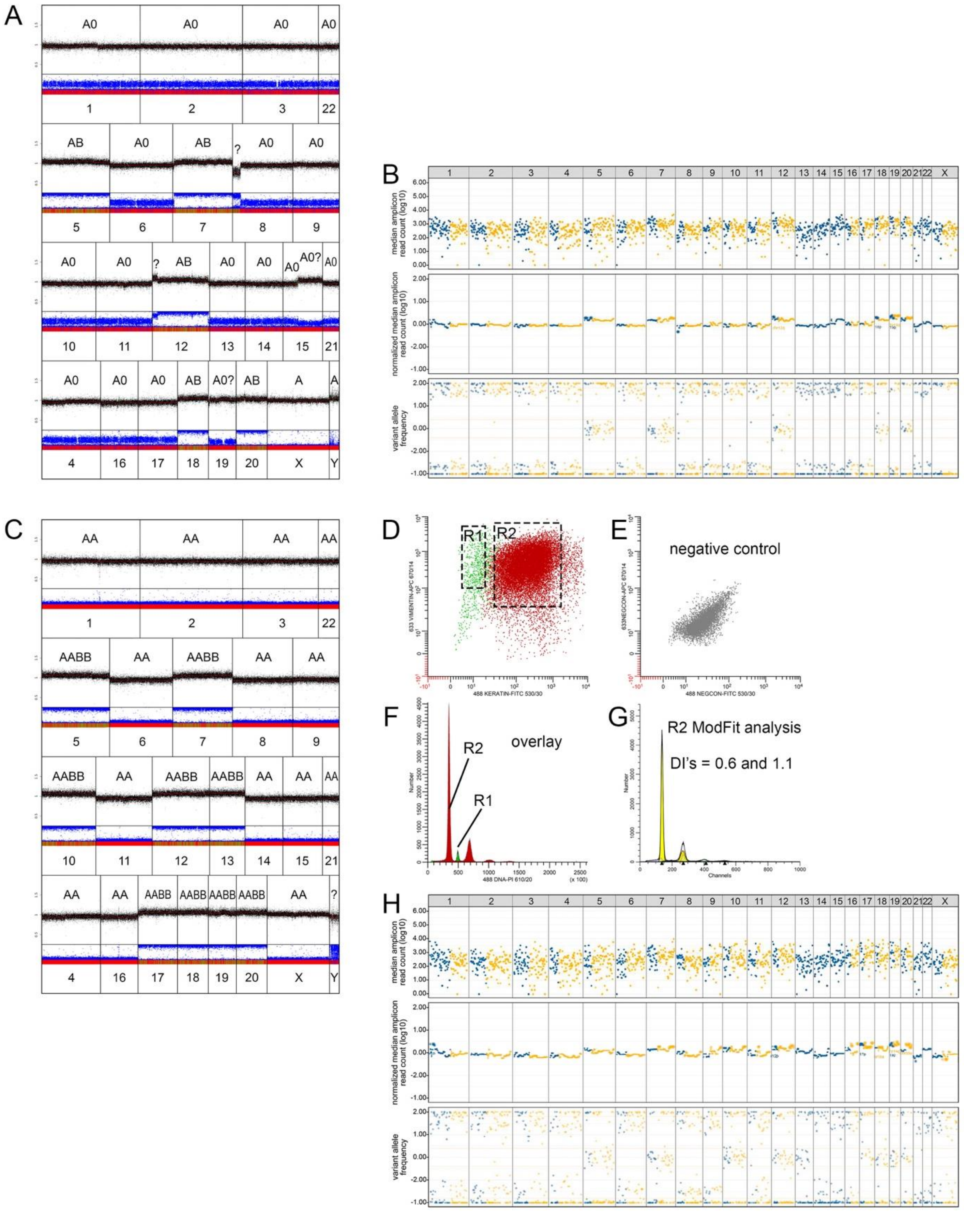

Verification of the CNA patterns observed during CNA-LOH analysis using the GWLOH v2 panel (**B and H**) using historical cases on which LAIR analysis (**A and C-G**) was previously performed according to the methods described by Corver et al. (Corver, *et al.* 2018). Two of the four historical cases are presented in Figure 3 of the main manuscript; here we present two more OCA. **A.** The SNP array analysis (iCOG and HumanCytoSNP-12, Illumina, Inc., San Diego, CA, USA) of one OCA in a male patient demonstrated CNA with homozygosity and chromosomal losses of chromosomes 1-4, 6, 8-11, 13-17, 19, 21, and 22. Unfortunately, no DNA content analysis by flow cytometry was performed for this case and exact information regarding the DNA index is lacking. Yet, based on the baseline of the SNPs (blue) on the iCOG SNP array, no endoreduplication is suspected. **B.** On CNA-LOH analysis using the GWLOH panel, this OCA also showed chromosomal losses of the affected chromosomes relative to the unaffected chromosomes (middle panel, normalized median amplicon read count). The VAF of the GWLOH analysis (bottom panel) of the affected chromosomes showed an SNP imbalance with a pronounced but not an extreme amplitude, consistent with GH type CNA. Based on the GWLOH panel, endoreduplication was considered possible, although the results were not highly suspicious for it. Altogether, results of the LAIR analysis are in accordance with the results of the GWLOH panel. The findings correspond best to GH type CNA without (suspected) endoreduplication.

C. The iCOG SNP array analysis of the other OCA in a male patient of this case showed homozygosity and chromosomal losses of chromosomes 1-4, 6, 8, 9, 11, 14-16, 21, and 22. **D.** Keratin (FITC) vs. vimentin (APC) fluorescence. Different populations R1 (vimentin-positive, keratin negative) and R2 (vimentin-positive and keratin-positive) can be observed. **E.** Negative control stained with the secondary reagents and PI only. **F.** Overlay of population R1 and R2. Note that the major G_0G_1-peak of the histogram is painted red and left to minor green G_0G_1-peak of R1, the DNA-diploid internal reference. This is indicative for loss of DNA. **G.** The DNA histogram of R2, the keratin-positive, vimentin-positive population showed to be bi-modal due to two cycling populations. In this case, the second G_0G_1 peak is significantly lower than that of the first G_0G_1 peak. The MFI of the R1 G_0G_1-population was used to accurately calculate the DNA index of the two G_0G_1 peaks of R2. The DNA indices were 0.6 and 1.1, respectively thus showing loss of DNA and near-haploidy and

endoreduplication. **H.** On the results of the GWLOH panel, chromosomal losses of the affected chromosomes relative to the unaffected chromosomes were observed (middle panel, normalized median amplicon read count), corresponding to GH type CNA. The SNP imbalances (bottom panel) showed high but noisy amplitudes. Endoreduplication was deemed possible, but assessment was difficult. The GWLOH results of this case illustrate that assessing the presence of endoreduplication using GWLOH analysis may be complex, especially in case of limitations regarding specimen quality and/or tumor cell percentage, but also in case of intra-tumor heterogeneity. The tumor cell percentage of the tested sample should always be considered when assessing the possible presence of endoreduplication. Altogether, for this OCA, the CNA pattern observed on the GWLOH panel was consistent with the findings of the LAIR analysis, too.

488 or 633, laser wavelength used for excitation. 530/30, band pass filter used to collect FITC fluorescence (green). 670/14, band pass filter used to collect APC fluorescence (infra-red). >610/20, long pass filter used to collect PI fluorescence (deep-red). APC, allophycocyanin. CNA, copy number alterations. CNA-LOH, copy number alterations and loss of heterozygosity. FITC, fluorescein isothiocyanate. GH type, genome haploidization type. GWLOH, genome-wide loss of heterozygosity. OCA, oncocytic thyroid carcinoma. LAIR, lesser-allele intensity-ratio. MFI, median fluorescence intensity. SNP, single nucleotide polymorphism. VAF, variant allele frequency.

References

Aydemirli MD, Snel M, van Wezel T, Ruano D, Obbink CMH, van den Hout WB, Schepers A & Morreau H 2021 Yield and costs of molecular diagnostics on thyroid cytology slides in the Netherlands, adapting the Bethesda classification. *Endocrinology, Diabetes & Metabolism*. (https://doi.org/10.1002/edm2.293)

Cohen D, Hondelink LM, Solleveld-Westerink N, Uljee SM, Ruano D, Cleton-Jansen AM, von der Thusen JH, Ramai SRS, Postmus PE, Graadt van Roggen JF, et al. 2020 Optimizing Mutation and Fusion Detection in NSCLC by Sequential DNA and RNA Sequencing. *J Thorac Oncol* 15 1000-1014. (https://doi.org/10.1016/j.jtho.2020.01.019)

Corver WE, Demmers J, Oosting J, Sahraeian S, Boot A, Ruano D, Wezel TV & Morreau H 2018 ROS-induced near-homozygous genomes in thyroid cancer. *Endocr Relat Cancer* 25 83-97. (https://doi.org/10.1530/ERC-17-0288)

Sibinga Mulder BG, Mieog JS, Handgraaf HJ, Farina Sarasqueta A, Vasen HF, Potjer TP, Swijnenburg RJ, Luelmo SA, Feshtali S, Inderson A, et al. 2017 Targeted next-generation sequencing of FNA-derived DNA in pancreatic cancer. *J Clin Pathol* 70 174-178. (https://doi.org/10.1136/jclinpath-2016-203928)

van der Tuin K, de Kock L, Kamping EJ, Hannema SE, Pouwels MM, Niedziela M, van Wezel T, Hes FJ, Jongmans MC, Foulkes WD, et al. 2019a Clinical and Molecular Characteristics May Alter Treatment Strategies of Thyroid Malignancies in DICER1 Syndrome. *J Clin Endocrinol Metab* 104 277-284. (https://doi.org/10.1210/jc.2018-00774)

van der Tuin K, Ventayol Garcia M, Corver WE, Khalifa MN, Ruano Neto D, Corssmit EPM, Hes FJ, Links TP, Smit JWA, Plantinga TS, et al. 2019b Targetable gene fusions identified in radioactive iodine refractory advanced thyroid carcinoma. *Eur J Endocrinol* 180 235-241. (https://doi.org/10.1530/EJE-18-0653)
